# How do children at special schools and their parents perceive their HRQoL compared to children at open schools?

**DOI:** 10.1186/1477-7525-8-72

**Published:** 2010-07-21

**Authors:** Jennifer Jelsma, Lebogang Ramma

**Affiliations:** 1 Division of Physiotherapy, School of Health and Rehabilitation Sciences, University of Cape Town, Cape Town, South Africa; 2 Division of Communication Sciences and Disorders, School of Health and Rehabilitation Sciences, University of Cape Town, Cape Town, South Africa

## Abstract

**Background:**

There has been some debate in the past as to who should determine values for different health states for economic evaluation. The aim of this study was to compare the Health Related Quality of Life (HRQoL) in children attending open schools (OS) and children with disabilities attending a special school (SS) and their parents in Cape Town South Africa.

**Methods:**

The EQ-5D-Y and a proxy version were administered to the children and their parents were requested to fill in the EQ-5D-Y proxy version without consultation with their children on the same day.

**Results:**

A response rate of over 20% resulted in 567 sets of child/adult responses from OS children and 61 responses from SS children. Children with special needs reported more problems in the "Mobility" and "Looking after myself" domains but their scores with regard to "Doing usual activities", "Pain or discomfort" and "Worried, sad or unhappy" were similar to their typically developing counterparts. The mean Visual Analogue Scale (VAS) score of SS children was (88.4, SD18.3, range 40-100) which was not different to the mean score of the OS respondents (87.9, SD16.5, range 5-100).

The association between adult and child scores was fair to moderate in the domains. The correlations in VAS scores between Open Schools children and female care-givers' scores significant but low (r = .33, p < .001) and insignificant between Special School children and adult (r = .16, p = .24).

**Discussion:**

It would appear that children with disabilities do not perceive their HRQoL to be worse than their able bodied counterparts, although they do recognise their limitations in the domains of "Mobility" and "Doing usual activities".

**Conclusions:**

This finding lends weight to the argument that valuation of health states by children affected by these health states should not be included for the purpose of economic analysis as the child's resilience might result in better values for health states and possibly a correspondingly smaller resource allocation. Conversely, if HRQoL is to be used as a clinical outcome, then it is preferable to include the children's values as proxy report does not appear to be highly correlated with the child's own perceptions.

## Introduction

The health of children is generally valued highly by society and is recognised as a priority for health service delivery by many organisations including the World Health Organisation. Prevention and management of diseases in children is one of the pillars of Primary Health Care and infant mortality is a well recognised marker of the health of a nation. In several studies, the health of children has been found to be valued more highly than the health of older people [1,2]. The health related quality of life of children is an important outcome measure for intervention [3] and is increasingly used as an outcome measure in conditions as diverse as lower urinary tract reconstruction in children with spina bifida[4], obesity [5] and tonsillectomy [6].

There has been some debate in the past as to whether the determination of values for different health states should include those with disabilities and those affected by the health states as valuers [7]. It has been found that people who have mild disability of adult onset show complete adaptation in all domains of life and that respondents with a severe disability of adult onset showed incomplete adaptation in only the health and income domains [8]. The inclusion of people with disabilities might therefore lead to an inflated value for health states relevant to their disabilities as they may perceive themselves to be less disabled than do the general public [9,10]. Whereas this is a desirable state of affairs, it might negatively impact resource allocation if such values are then used in cost-utility analysis. There is less evidence regarding the perception of HRQoL of children with functional limitations, but the few studies that have been done, report contrasting findings. A qualitative study on children with cerebral palsy reported that on a scale from 1 to 10, most of the twelve adolescents rated their life as eight or above[11], which would appear to be quite high. In contrast, children with meningomyocele reported significantly lower quality of life than the US norms[12].

Generally, proxy measures are used when the respondent is unable to answer on his/her own behalf, e.g. in cases of incapacitation or incompetence [13]. The description and valuation of a child's health state has generally been based on the proxy report of the principal care-givers[14], which has been reported to be feasible and valid within a population of between 1 and 15 years of age [15,4]. A problem that Lara and Badia identified during a literature review of the use of proxy responses was that papers were not specific as to the perspective from which the proxies reported the HRQoL of the subjects, i.e. whether they were asked to report on their perception of the subjects health state or what they estimated would be the subjects description of his/her health state if they were to answer for themselves [13]. In addition, proxy measures are often used without adequate interrogation of whether the responses truly represent the view of the child [12,16].

The EQ-5 D is an instrument that has been used extensively in adults to gather information related health related quality of life (HRQoL). It does not attempt to examine the broader concept of quality of life but is restricted to dimensions related in some way to health. It consists of a section which collects descriptive data about HRQoL and a section which gathers self-rating of current health state[17]. In 2007, the EQ-5D-Y version which was developed expressly for use in children was accepted as the definitive version of the EQ-5 D to be used with children. This has been subject to an international process to establish reliability and validity[18,19] and has been found to be a valid instrument to measure HRQoL in children eight years and older[20]. The EQ-5D-Y consists of five domains of functional impairment; "Mobility", "Looking after myself", "Doing usual activities", "Pain or discomfort" and "Worried, sad or unhappy". The respondent has the option of reporting no problems, some problems or severe problems in each of these domains. Each participant is required to fill in a visual analogue scale (VAS) which ranges from 0, worst health state imaginable to 100, best health state imaginable. The health state may be regarded as the objectively observed state of the respondent whereas the VAS reflects self-assessment of this state. It is unclear whether the objective and subjective assessment of health state are similar in children with disabilities.

The study set out to examine several related issues. Do children with functional limitations perceive their HRQoL to be worse than do children attending open schools? Are proxy responses given by care-givers a valid indication of the HRQoL of their children who have functional limitations? What factors, including problems in functional domains, gender and attendance at a SS determine the VAS score of children? The specific objectives were, with regard to the current health state of the child,:

◦ To determine whether there was a difference in self-reported HRQoL between children attending a Special School (SS) and children attending an Open School (OS).

◦ To establish whether the descriptor state, the age, gender or attendance at a SS are determinants of the self-reported HRQoL of the child as measured by the VAS.

◦ To determine if the description and perception of HRQoL differ between children and their parents

It was anticipated that the presence of problems on the descriptor domains ("Mobility", "Pain or discomfort" etc.) would reduce the VAS score. What was less clear was whether the presence of a functional limitation severe enough to warrant attendance at a SS would in itself result in a decrease in score.

## Methodology

A cross-sectional descriptive analytical study design was utilised.

In Cape Town, children with special needs attend schools which provide therapeutic and remedial services. The school that participated in this study provides schooling for children with a range of functional impairments, ranging from learning disabilities to movement disorders. Admission to this school is based on the child's ability to follow the conventional school curriculum and children with severe learning difficulties would be referred to another specialised school.

There were two samples recruited to the study. The first consisted of children attending primary schools in the Cape Town area. In South Africa, children start school the year that they turn seven so that the ages of the respondents would range from approximately 7 to 12 years of age. Two single sex schools from an advantaged area (median income between $300 and $550 per month) and two co-educational schools from a relatively socio-economically deprived area (median income less that $300 per month) were chosen for the study. The second group of respondents was recruited from the primary school section of a co-education school catering to educable children with special needs. All children who were present on the day of the study and who met the study requirements of parental consent and parental participation were included in the study. There were no exclusion criteria and children who were unable to physically fill in the forms themselves were assisted by the research assistants.

### Instrumentation

The EQ-5D-Y was administered to all children. This is a recently developed instrument which was developed under the auspices of the EuroQol Foundation. It has been found to be valid measure of HRQoL in children in Cape Town[21] and elsewhere [19].The EQ-5D-Y proxy version which requests that the adult respondent answer as he/she would expect the child to respond was used (as opposed to asking the proxy to rate the child's health from the proxy's perspective).

### Procedure

Ethical approval to conduct the study was received from the Medical Research Ethics Committee of the University of Cape Town and from the Department of Education. Children in the eligible grades were each given consent forms to take home for completion by their parents/caregivers. The children who returned these forms and who gave assent to the study were given 10-15 minutes to complete the questionnaire in the presence of at least one of the researcher assistants. An explanation of what was required was given and all pupils were allowed to ask for clarification if necessary.

On collection of the completed pupil questionnaires, the respondents were given proxy questionnaires and an information sheet to take home to their parents. The questionnaires and the consent and the assent forms were coded according to the school, grade and class, which assured anonymity.The parents were requested not to consult with each other or their child before filling in the proxy version. In addition they were requested to fill in the proxy version on the same day as their child had filled in the EQ-5D-Y.

Five children at the special needs school needed the assistance of a helper to fill out the form as they were incapable of doing it themselves. In these cases, it was made clear that the answers were to be given by the child and not by the helper.

### Statistical analysis

Descriptive statistics were used to describe the demographics of the sample and the health state of child as described by the children. As there were few respondents who reported severe problems, the categories "some" and "lots" of problems were collapsed and the Kappa statistic was used to determine the percentage of agreement between adults and child. Pearson's correlation co-efficient was determined to examine the correlation between the VAS scores of the different sets of respondents. Multiple regression analysis was used to determine which variables were predictive of the child's perceived health status. These variables included grade and dummy variables which were created for gender, attendance at a special school and presence of a problem in one of the five domains. All variables were entered simultaneously and preliminary residual analysis was done.

## Results

In open schools, 567 primary school learners in total took part, of which 253 were male (45%). In the special needs school, there were 61 respondents of which 45 (74%) were male. There was no difference in the percentage of questionnaires returned from the two settings (28.2% for SS and 28.4% for SS). All grades were represented with the largest number (29%) in Grade 4 in the open schools and in Grade 6 in the Special School (31%).

Children from Open Schools reported the most problems in the "Pain or discomfort" domain, whereas the children from the Special School had most problems in the "Mobility" domain (Table 1). The distribution between the two groups was significantly different in the "Mobility" and "Looking after myself" domains, with the Special School children reporting more problems. In the other three domains children from the Special School reported less problems but the difference was not statistically significant

**Table 1:** Comparison of Open and Special School responses to the different domains (n = 62, 5 missing responses in total)

Domain	No Problems Frequency (%)	Some Problems Frequency (%)	A lot of Problems Frequency (%)	Missing Answers Frequency (%)	Chi Sq (p value)
**"Mobility"**					

Open School	525 (92.6)	37 (6.5)	5 (0.9)		18.1 (<.001)

Special School	47 (77.0)	11 (18.0)	3 (4.9)		

**"Looking after myself"**					

Open School	547 (96.5)	20 (3.5)	0		15.1 (<.001)

Special School	54 (88.5)	6 (9.8)	1 (1.6)		

**"Doing usual activities"**					

Open School	489 (86.2)	75 (13.2)	2 (0.4)	1 (0.2)	3.1 (.21)

Special School	55 (90.2)	5 (8.2)	1 (1.6)		

**"Having pain or discomfort"**					

Open School	395 (68.7)	162 (28.6)	9 (1.6)	1	4.2 (.13)

Special School	50 (82.0)	10 (16.4)	1 (1.6)	1 (0.2)	

**"Feeling worried, sad or unhappy"**					

Open School	409 (72.1)	148 (26.1)	8 (1.4)	2 (0.4)	2.9 (.23)

Special School	48 (78.6%)	11 (18.0%)	2 (3.3%)	0	

The mean VAS of the Open School respondents was 87.9 (SD 16.5, range 5-100) which was not different to the mean score of the children from the Special School (88.4, SD 18.3, range 40-100)

**Figure 1:**
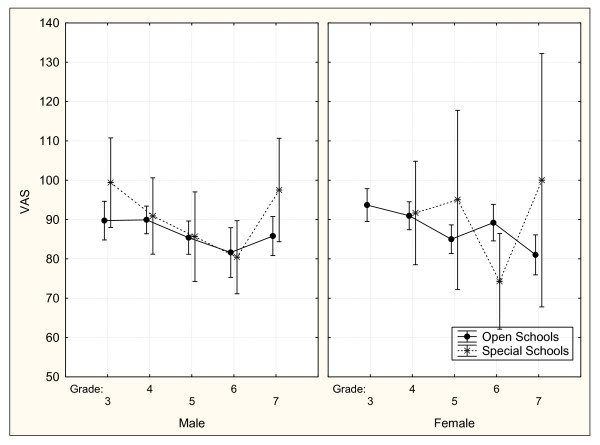
**VAS scores by gender, grade and type of school**. Vertical bars denote 95% confidence intervals.

The VAS across gender, grade and school type is depicted in Figure 1. There is a general trend toward decreasing scores with increasing grade. The male results from the OS and SS follow each other quite closely but the female scores show more variation. Apart from the Grade 6 respondents, children at SS reported an equal or better health state that the OS respondents. These relationships were examined further using multiple regression analysis as described below.

The determinants of the child's VAS were examined and a model was developed which included gender, grade, attending Special School and the presence of problems in each dimension (Table 2). The model did not fit the data well and only accounted for 13% of the variance and there were 22 participants whose predicted scores fell more than two standard deviations away from their observed scores. Gender and attendance at a Special school did not predict the VAS, whereas VAS decreased significantly by 1.5 for each grade, and by 5.9, 5.0 and 4.7 for a problem reported in "Doing usual activities", "Pain or discomfort" and "Worried, sad or unhappy" respectively.

**Table 2:** Predictors of child's VAS - All children (n = 611, some missing data)

	B	Std Error of B	t(611)	p-level
**Intercept**	73.7	4.39	16.8	0.00

**Open School**	0.4	2.16	0.2	0.87

**Female**	0.9	1.26	0.7	0.48

***Grade***	*-1.5*	*0.49*	*-3.1*	*0.00*

**"Mobility" problem**	-3.8	2.40	1.6	0.11

**"Looking after myself" problem**	-6.0	3.20	1.9	0.06

***"Doing usual activities"problem***	*-5.9*	*1.96*	*3.0*	*0.00*

***"Having pain or discomfort" problem***	*-5.0*	*1.47*	*3.4*	*0.00*

***"Feeling worried, sad or unhappy" problem***	*-4.7*	*1.47*	*3.2*	*0.00*

R^2 ^= .13 Italics denote significance.

### Comparison of children and adult scores

There were 530 female adult respondents from the Open Schools Group and 57 from the Special School Group (6% missing in both cases) compared to 495 and 35 male respondents respectively (11 and 57% missing respectively). As the Kappa level of agreement was the same between male and female parents for all domains except for "Doing usual activities" (Females Slight compared to Males in Fair Agreement in the Open Schools sample) only the adult female responses are presented. Table 3 indicates that generally there was greater agreement between children at Special Schools and their female care-givers in terms of the problems that they reported.

**Table 3:** Agreement between parents and children in each domain of the EQ5 D Questionnaire using Cohen's Kappa, in both socio-economic groups.

Domain	Child/mother Kappa Open Schools		Child/mother Kappa Special School	
**"Mobility"**	K = 0.15 Slight Agreement	6.2**% **Child More .5% Adult More	K = .60 Moderate Agreement	5.3**% **Child More 10.5.% Adult Morr

**"Looking after myself"**	K = 0.08 Slight Agreement	3.2**% **Child More 5.3.% Adult More	K = .33 Fair Agreement	1.8**% **Child More 17.5.% Adult More

**"Doing usual activities"**	K = 0.01 Slight Agreement	10.5**% **Child More 6.4% Adult More	K = .34 Fair Agreement	1.8**% **Child More 17.5% Adult More

**"Having pain or discomfort"**	K = 0.20 Slight Agreement	19.4**% **Child More 11.7% Adult More	K = .41 Moderate Agreement	5.3**% **Child More 15.8% Adult More

**"Feeling worried, sad or unhappy"**	K = 0.21 Fair Agreement	15.1**% **Child More 16.8% Adult More	K = .22 Fair Agreement	8.8**% **Child More 17.5% Adult More

("Some" and "Lots of Problems" were collapsed into a problem category). The second columns indicate the % of child and adult respondents who reported more problems than the other member of the dyad.

The correlation in VAS scores between Open Schools children and female care-givers' scores on the VAS were significant but low (r = .33, p < .001) and insignificant between Special School children and adult (r = .16, p = .24) The correlation between the male and female care-givers was r = .66 (p < .001) for Open School children and similar, r = .67 (p < .001) for the Special School children.

The mean value of the female care-givers' VAS scores for Open School respondents was 90.4 (SD12.3) which was significantly more that the children's own score of 88.4 (SD15.7, p = .006). In contrast the mean score of the Special School adult respondents 85 (SD15.8) was less than the children's but this was not significant.

## Discussion

The sample was representative of the two groups and the final response rate indicated little difference between the Open and Special Schools samples. There were more females in the open schools and more males in the special school but as multivariate analysis indicated that gender did not predict the VAS of the child, this should not have biased the results. Each grade was represented by at least 10% of the sample, although the number of children in Grades 1 and 7 in the Special School was small.

The most striking finding of this study was that, although children attending SS appeared to recognize that they had functional limitations (as evidenced by reporting more problems in the domains), this did not translate into a perception of lower HRQoL (as measured by the VAS). This finding is similar to Liu et al (2009) who concluded that gross motor functions may be good predictors of the physical component of health-related quality of life, but they are poor predictors of the psychosocial component of health-related quality of life in children with cerebral palsy[16]. In fact the children in this group seemed to be remarkably resilient and reported a VAS score that was higher than children attending open schools. Although they reported more problems in the "Mobility" and "Looking after myself" domains, as would be expected, the number reporting problems with pain or with anxiety was no greater than children at OS. This resilience was noted in a study of children with spina bifida in Kenya which noted that although their HRQoL was lower than that of healthy controls, it 'remains surprisingly acceptable'[22]. In addition the children perceived themselves to have fewer problems than reported on their behalf by their female care-givers, despite the proxies being requested to answer as they thought the child might respond.

The EQ-5D-Y performed well and there were few missing responses which would indicate that the EQ-5D-Y can be validly used in this age group, a finding supported by other studies [19,23]. The frequency distribution of the problems encountered in every domain in the Open Schools is similar to regional studies of adults[24] and children[23] using the EQ-5 D and EQ-5D-Y in that "Pain or discomfort" and "Worried, sad or unhappy" are the areas in which problems are most commonly reported. The results from the Special School reflect the entrance criteria for that school which include physical disabilities and learning problems and the respondents from Special Schools did report significantly more problems in the areas of "Mobility" and "Looking after myself".

A qualitative study on QoL in children with cerebral palsy reported that pain and restricted mobility and accessibility were the factors related to CP that contributed to a lower QoL but the disability itself was typically not viewed as an important factor contributing to QoL [11]. Similarly this study found that attendance at a Special School was not predictive of a child's perceived VAS. The validity of the EQ-5D-Y was supported in that in the Open Schools sample, the presence of problems in the different domains was the strongest predictor of VAS, with each domain detracting a similar amount from the VAS score. As the Special School sample did not report poorer HRQoL, the impact of "Mobility" and "Looking after myself" problems was not significant in the entire group. As noted in other studies[5], adolescents report a poorer HRQoL than younger children and the VAS did decrease as the respondents moved into the higher grade. The differential impact of higher SES income was lost in the multiple regression analysis, possibly because of the large number in this group reporting "Pain or discomfort" and "Worried, sad or unhappy" problems

As expected, a larger number of female adult respondents returned proxy versions but it is unclear if the number of missing adult responses (6% female and 11% male) were due to children residing in single parent households or simply due to lack of response compliance. It is assumed that in most cases the female adult was the mother and the male adult was the father but the exact relationship to the child was not asked in the questionnaire. The number of questionnaires returned by parents was lower than anticipated (20%) but post-hoc analysis indicated that there was no difference in the VAS score and the number of children with disabilities between the defaulters and the other children. If bias was introduced, it was not detected by this analysis.

There was a general trend for the adult respondents of the Open School children to report better HRQoL for their children than the children themselves. In contrast the adults reported worse HRQoL than their children in the Special School, which again highlights the resilience of children with long term functional problems. The issue of discordance between child and parent proxy report has been identified as a problem in cost-utility analysis [25] and the, at best, moderate percentage agreement on the descriptor domains and low correlation between care-givers and children bears this out. The satisfactory correlation between the female and male care-givers would indicate that, provided proxy and child respondent reports are not used interchangeably, proxy reports appear to be reliable.

## Conclusions

Children attending special schools did not perceive their health state to be worse than their peers at open schools. This finding lends weight to the argument that valuation of chronic health states by children affected by these health states should not be included for the purpose of economic analysis as the child's resilience might result in better values for health states. This might result in a correspondingly smaller resource allocation and it is suggested that if an objective measure of the child's health state is required for, e.g. evaluation of functioning to estimate need of extra resources, an adult proxy measure is preferable. Conversely, if HRQoL is to be used as a clinical outcome, then it is advisable to include the children's subjective values as proxy report does not appear to be highly correlated with the child's own perceptions.

The use of the proxy version yields useful but somewhat different information and seems to be a reliable method of obtaining information about the HRQoL of children as there is good agreement between care-givers with regard to their child. However the proxy and the self-report versions should not be used interchangeably as they do not give the same information.

## Competing interests

The authors declare that they have no competing interests.

## Authors' contributions

JJ conceptualized the project and gathered the data. JJ and LR contributed to the write-up and revision of the final manuscript.
